# How cardiologists manage antithrombotic treatment of patients with atrial fibrillation undergoing percutaneous coronary stenting: the WOEST survey 2018

**DOI:** 10.1007/s12471-020-01500-3

**Published:** 2020-10-14

**Authors:** A. J. W. M. de Veer, N. Bennaghmouch, W. J. M. Dewilde, J. M. ten Berg

**Affiliations:** 1 Department of Cardiology, St. Antonius Hospital, Nieuwegein, The Netherlands; 2 Department of Cardiology, Imelda Hospital, Bonheiden, Belgium

**Keywords:** Anticoagulation, Antiplatelet therapy, Acute coronary syndrome, Percutaneous coronary intervention, Atrial fibrillation, Non-vitamin K antagonist oral anticoagulants

## Abstract

**Background:**

Antithrombotic treatment choices are complicated when patients have both atrial fibrillation (AF) and acute coronary syndrome and/or undergo percutaneous coronary intervention (PCI). In this study, we aimed to gain insight into antithrombotic management strategies in daily clinical practice.

**Methods:**

We invited interventional cardiologists to complete the WOEST (What is the Optimal antiplatElet & Anticoagulant Therapy in Patients With Oral Anticoagulation and Coronary StenTing) survey 2018. In this questionnaire, we presented a patient with a non-ST-elevation myocardial infarction (NSTEMI) and an elective PCI case.

**Results:**

The results were based on 118 completed questionnaires (response rate 69.4%). In the case of the AF patient with NSTEMI, most cardiologists indicated they would initiate dual antiplatelet therapy (acetylsalicylic acid and clopidogrel) and continue non-vitamin K antagonist oral anticoagulant (NOAC) therapy at admission and during coronary angiography/PCI. At discharge, 70.3% would prescribe triple antithrombotic therapy (oral anticoagulation, acetylsalicylic acid and clopidogrel), mostly for 1 month. One year after NSTEMI, 83.1% would cancel the antiplatelet therapy and prescribe NOAC monotherapy. For the AF patient undergoing elective PCI, 51.7% would start dual antiplatelet therapy prior to the procedure and 52.5% would discontinue NOAC therapy prior to the PCI. At discharge, 55.1% would start triple antithrombotic therapy. Furthermore, 25.4% responded they routinely prescribe a reduced dose of NOAC after discharge. One year after PCI, 89.0% would continue NOAC monotherapy.

**Conclusion:**

The WOEST survey demonstrated heterogeneity in antithrombotic management strategies among interventional cardiologists. This observed variety mirrors the heterogeneity of the many guidelines and consensus documents. Further research is needed to guide patient-tailored medicine for AF patients undergoing PCI.

**Electronic supplementary material:**

The online version of this article (10.1007/s12471-020-01500-3) contains supplementary material, which is available to authorized users.

## What’s new?

- To investigate antithrombotic management strategies for atrial fibrillation (AF) patients treated with oral anticoagulation undergoing percutaneous coronary intervention (PCI) with stenting, we carried out a survey among interventional cardiologists.
- Dual antiplatelet therapy was the most prescribed antithrombotic regimen for AF patients admitted to the hospital with non-ST-elevation myocardial infarction.
- One year after PCI, most of the cardiologists (>80%) would cancel the antiplatelet therapy and continue monotherapy with a non-vitamin K antagonist oral anticoagulant.

## Introduction

For patients with atrial fibrillation (AF) and an increased CHA_2_DS_2_-VASc score (≥2 for men and ≥3 for women), guidelines recommend lifelong treatment with oral anticoagulation (OAC) to reduce stroke risk [1]. For patients with acute coronary syndrome (ACS) or undergoing percutaneous coronary intervention (PCI) with stenting, guidelines recommend treatment with dual antiplatelet therapy (DAPT), consisting of acetylsalicylic acid and a P2Y_12_ inhibitor to reduce the risk of stent thrombosis and other atherothrombotic events [2, 3]. However, treatment choices are complicated when patients have both AF and ACS and/or undergo PCI. Current clinical guidelines acknowledge considerable gaps in the availability of high-quality evidence regarding optimal antithrombotic therapy for these patients.

Recently, the results of four randomised controlled trials have been published, in which the effect of vitamin K antagonists (VKAs) versus non-vitamin K antagonist oral anticoagulants (NOACs) in combination with antiplatelet therapy was investigated in patients undergoing PCI with stenting [4–7]. Furthermore, European and American expert consensus documents have been updated [8, 9]. According to the European guidelines, most patients should be treated with both OAC and DAPT (i.e. triple antithrombotic therapy). However, the inevitable trade-off of intensification of antithrombotic therapy are more bleeding complications, which is associated with increased mortality [10–12]. Therefore, antithrombotic treatment for this complex population should be carefully balanced.

We have noticed important differences in antithrombotic strategies carried out by cardiologists. To obtain insight into the current antithrombotic strategies in daily clinical practice for patients with both AF and ACS and/or undergoing PCI with stenting, we carried out a survey in Dutch and some international PCI centres.

## Methods

The WOEST (What is the Optimal antiplatElet & Anticoagulant Therapy in Patients With Oral Anticoagulation and Coronary StenTing) survey 2018 is a questionnaire addressing the antithrombotic treatment of patients with AF undergoing PCI with stenting in daily practice from the perspective of the treating interventional cardiologist. This survey was based on a 19-item online questionnaire sent out to all interventional cardiologists working in the 27 PCI centres in the Netherlands and to individual interventional cardiologists in 24 PCI centres in Belgium, Germany, the United Kingdom, Denmark, Austria, Switzerland, France, Spain, Italy, Greece, Portugal and Sweden. The survey was conducted from September to December 2018.

As an introduction to the questions posed, we presented two cases: (1) AF patient using a NOAC who is hospitalised for a non-ST-elevation myocardial infarction (NSTEMI) and needs urgent PCI, and (2) AF patient using a NOAC who is diagnosed with chronic coronary syndrome and needs to undergo elective PCI. Both patients carry an intermediate bleeding and thrombotic risk. A complete overview of the survey questions is shown in Tab. 1 (see Electronic Supplementary Material). The survey was reviewed by an expert panel.

The Medical research Ethics Committees United confirmed that the Medical Research Involving Human Subjects Act (*Wet medisch-wetenschappelijk onderzoek met mensen*) did not apply to this noninterventional study; hence, official ethical approval by the ethics committee was not required. Declaration approval was obtained from the Institutional Review Board. A local study protocol is available. The study was conducted in accordance with the Declaration of Helsinki.

### Statistical analysis

Descriptive analysis of the data was performed using summary statistics for categorical and quantitative (continuous) data. Categorical data are expressed as frequencies with percentages. Statistical analysis was performed using SPSS software for Windows, version 24 (IBM Corporation, Armonk, NY, USA).

## Results

### Participants

A total of 118 interventional cardiologists completed the questionnaire (response rate 69.4%). Of them, 100 cardiologists (84.7%) worked in a total of 27 different PCI centres in the Netherlands.

### Outcomes

Of the responding cardiologists, 73.7% reported that a standard local protocol is available for the antithrombotic management of patients on OAC who require PCI.

For the AF patient with NSTEMI, 95.8% of the cardiologists would start antiplatelet therapy as soon as possible after admission. The majority of these cardiologists (70.3%) responded they initiate DAPT (acetylsalicylic acid and clopidogrel), 22.9% initiate P2Y_12_ inhibitor monotherapy (ticagrelor 90 mg twice daily, clopidogrel 75 mg once daily or prasugrel 10 mg once daily), and 6.8% initiate acetylsalicylic acid monotherapy. More than half (56.8%) would continue NOAC therapy at admission and during coronary angiography/PCI. During PCI, 33.9% would administer a reduced dose of heparin and 3.4% chose not to administer heparin at all.

At discharge, most of the cardiologists (70.3%) would treat a patient with an intermediate bleeding risk as well as an intermediate thrombotic risk with triple antithrombotic therapy. In case of a high bleeding risk, 9.3% would start triple therapy; in case of both a high bleeding and a high thrombotic risk, 43.2% would initiate this treatment. When prescribing triple antithrombotic therapy, the majority (83.1%) would do this for 1 month. A reduced dose of a NOAC (rivaroxaban 15 mg once daily, apixaban 2.5 mg twice daily, dabigatran 110 mg twice daily in patients <80 years, edoxaban 30 mg once daily) would be prescribed by 34.7% of the cardiologists; the others would prescribe the routinely dosed NOAC. After 1 year, the majority (83.1%) would cancel the antiplatelet therapy and prescribe NOAC monotherapy.

For the AF patient undergoing elective PCI, 51.7% would start DAPT prior to elective PCI. The others would start single antiplatelet therapy, mainly a P2Y_12_ inhibitor. More than half (52.5%) indicated to discontinue NOAC therapy prior to PCI and no one chose to bridge the anticoagulation therapy. If NOAC therapy is continued during PCI, 4.2% would not administer heparin during the procedure, which means that 95.8% *would* give heparin, one-third of whom would use a reduced dose.

At discharge, the default strategy was triple antithrombotic therapy for more than half of the cardiologists (55.1%); the others would start dual antithrombotic therapy consisting of a P2Y_12_ inhibitor and an oral anticoagulant. In case of a high bleeding risk, 5.1% would start triple antithrombotic therapy; in case of both a high bleeding and a high thrombotic risk, 31.4% would start this treatment. When triple antithrombotic therapy is prescribed, 89.8% would prescribe acetylsalicylic acid for 1 month. Furthermore, 25.4% indicated to prescribe a reduced dose of NOAC after discharge. One year after PCI, 89.0% would stop the antiplatelet therapy and continue NOAC monotherapy.

In the elective PCI setting, 38.1% of the cardiologists would switch to a NOAC if the patient is using a VKA. If the patient has ACS, is using a VKA and is in urgent need of PCI, 37.3% would switch to a NOAC. All results are shown in Tab. 1.

**Table 1:** Management of AF patients on NOAC therapy undergoing PCI, according to the WOEST survey 2018 among 118 cardiologists

Question	Answer	*n* (%)	
*General*			
Is there a standardised protocol for the antithrombotic management of AF patients on VKA or NOAC who require PCI?	Yes, but only for patients VKA	4 (3.4)	
	Yes, for patients on chronic VKA and NOAC	87 (73.7)	
	No, there is no protocol	27 (22.9)	
*Before PCI*		*NSTEMI*	*Elective PCI*
Would you initiate antiplatelet therapy immediately after admission?	Yes	113 (95.8)	N/A
	No, I would start this during/after PCI	5 (4.2)	N/A
If yes, which antithrombotic regimen would you start?	ASA monotherapy (+NOAC)	8 (6.8)	N/A
	P2Y_12_ inhibitor monotherapy (+NOAC)	27 (22.9)	N/A
	Dual antiplatelet therapy (+NOAC)	83 (70.3)	N/A
Would you discontinue the NOAC therapy before PCI?	Yes, without bridging	43 (36.4)	62 (52.5)
	Yes, with bridging	8 (6.8)	0
	No	67 (56.8)	56 (47.5)
*During PCI*		*NSTEMI*	*Elective PCI*
Would you administer a bolus of heparin (UFH/LMWH) at the start of PCI when the patient is on NOAC?	Yes, the standard dose	74 (62.7)	78 (66.1)
	Yes, a reduced dose	30 (33.9)	35 (29.7)
	No	4 (3.4)	5 (4.2)
*After PCI*		*NSTEMI*	*Elective PCI*
At discharge, what is your default antithrombotic strategy for a patient like this (intermediate bleeding risk and intermediate thrombotic risk)?	Triple therapy	83 (70.3)	65 (55.1)
	Dual therapy	35 (29.7)	53 (44.9)
In case of a *high bleeding risk*, what is your default antithrombotic strategy at discharge?	Triple therapy	11 (9.3)	6 (5.1)
	Dual therapy	107 (90.7)	112 (94.9)
In case of a *high bleeding risk *and *a high thrombotic risk*, what is your default antithrombotic strategy at discharge?	Triple therapy	51 (43.2)	37 (31.4)
	Dual therapy	67 (56.8)	81 (68.6)
When you use triple therapy, for what period of time would you prescribe ASA?	1 month	98 (83.1)	106 (89.8)
	3 months	8 (6.8)	4 (3.4)
	6 months	7 (5.9)	3 (2.5)
	12 months	4 (3.4)	3 (2.5)
	>12 months	1 (0.8)	2 (1.7)
Which NOAC for stroke prevention would you prescribe at discharge?	I would continue the same NOAC	93 (78.8)	97 (82.2)
	Dabigatran	10 (8.5)	10 (8.5)
	Rivaroxaban	2 (1.7)	1 (0.8)
	Apixaban	11 (9.3)	8 (6.8)
	Edoxaban	2 (1.7)	2 (1.7)
Which dose would you prescribe at discharge?	The normal dose	77 (65.3)	88 (74.6)
	The reduced dose	41 (34.7)	30 (25.4)
If the patient was using a VKA instead of a NOAC, would you switch the patient to a NOAC after PCI?	Yes	44 (37.3)	45 (38.1)
	No	74 (62.7)	73 (61.9)
Which antithrombotic treatment would you prescribe >1 year after PCI?	Monotherapy: NOAC	98 (83.1)	105 (89.0)
	Dual therapy: NOAC and ASA or P2Y_12_ inhibitor	20 (16.9)	2 (11.0)

*AF* atrial fibrillation, *NOAC* non-vitamin K antagonist oral anticoagulant, *PCI* percutaneous coronary intervention, *VKA* vitamin K antagonist, *NSTEMI* non-ST-elevation myocardial infarction, *ASA* acetylsalicylic acid, *UFH/LMWH* unfractionated heparin/low-molecular weight heparin, *N/A* not applicable

## Discussion

The WOEST survey 2018 provides insight into daily clinical practice with regard to antithrombotic therapy in AF patients with ACS and/or undergoing PCI in European PCI centres. We observed a large heterogeneity in management strategies among the interventional cardiologists.

The recent guidelines from the European Society of Cardiology (ESC) on the management of patients with AF favour the use of NOACs over VKAs in patients with nonvalvular AF [1]. As approximately 20% of AF patients require PCI at some stage, many patients need additional antiplatelet therapy [13]. However, there is currently no clear guideline on the management of NOACs in patients undergoing PCI, similar to when these patients are using a VKA. This could explain why >40% of the cardiologists in our survey would discontinue NOAC therapy in patients with NSTEMI undergoing PCI. In line with this observation, 53% of the cardiologists would discontinue the NOAC before performing an elective PCI. This indicates a reluctance to treat patients with PCI when they are on NOAC therapy.

This reluctance might be explained by the lack of evidence for the safety of NOACs in the catheterisation laboratory and the heterogeneity in practice guideline recommendations for these patients. In a small randomised phase IIa study, more thrombotic events occurred in patients treated with dabigatran (direct thrombin inhibitor) during elective PCI than in those treated with unfractionated heparin [14]. As for rivaroxaban (direct factor Xa inhibitor), the X‑PLORER study has shown that rivaroxaban (with or without unfractionated heparin) effectively inhibits coagulation activity during elective PCI in patients with chronic coronary syndrome compared with unfractionated heparin alone [15]. Furthermore, the OASIS‑5 trial has shown that the use of fondaparinux (factor Xa inhibitor) results in more catheter-related thrombosis than treatment with enoxaparin (low-molecular weight heparin) [16]. This may be one of the reasons why some interventional cardiologists are less confident in the performance of NOACs in the catheterisation laboratory.

The small WOEST trial was the first to compare the combination of a VKA plus clopidogrel to conventional triple therapy (consisting of VKA, clopidogrel and acetylsalicylic acid) and has shown that cancelling acetylsalicylic acid decreases the incidence of bleeding events in AF patients undergoing PCI without an increase in the incidence of thromboembolic events [17]. Following the WOEST trial, four international randomised trials have been conducted investigating the role of a NOAC-based antithrombotic strategy versus conventional triple therapy. In the PIONEER AF-PCI trial (A Study Exploring Two Strategies of Rivaroxaban and One of Oral Vitamin K Antagonist in Patients With Atrial Fibrillation Who Undergo Percutaneous Coronary Intervention), two rivaroxaban-based treatment regimens significantly reduced bleeding complications following PCI compared with conventional triple therapy, without increasing thromboembolic complications [4] Of note, the tested rivaroxaban dose (15 mg once daily) differed from the dose used in the AF trial (20 mg once daily). Dual therapy with dabigatran also reduces bleeding complications, as shown in the REDUAL-AF PCI trial (Evaluation of Dual Therapy With Dabigatran vs. Triple Therapy With Warfarin in Patients With AF That Undergo a PCI With Stenting) [5].

The AUGUSTUS trial (An Open-label, 2 × 2 Factorial, Randomised Controlled, Clinical Trial to Evaluate the Safety of Apixaban vs. Vitamin K Antagonist and Aspirin vs. Aspirin Placebo in Patients with Atrial Fibrillation and Acute Coronary Syndrome or Percutaneous Coronary Intervention) is the only trial that compared NOAC versus VKA plus DAPT and NOAC versus VKA with single antiplatelet therapy in a 2 × 2 design. It has shown that acetylsalicylic acid (aspirin), compared with placebo, is associated with significantly more bleeding events, but that the use of apixaban, compared with VKA, reduces the number of bleeding events [6]. Furthermore, in the ENTRUST-AF PCI trial (Edoxaban Treatment Versus Vitamin K Antagonist in Patients With Atrial Fibrillation Undergoing Percutaneous Coronary Intervention), edoxaban dual antithrombotic therapy was noninferior to triple antithrombotic therapy with respect to major or clinically relevant nonmajor bleeding [7].

Finally, recent meta-analyses have shown that in patients with AF undergoing PCI, dual antithrombotic therapy (either NOAC or VKA in combination with single antiplatelet therapy) reduces the composite of a TIMI (thrombolysis in myocardial infarction) major or minor bleeding by 47% as well as intracranial haemorrhages [18, 19]. Although the four trials were not powered to detect differences in thromboembolic events [4–7]; in none of the studies, this endpoint was significantly increased in the patients treated with dual therapy.

Evaluating all available data, one might argue that most evidence points to a NOAC-based regimen of dual therapy. Conversely, the current ESC guidelines (last updated before the AUGUSTUS and ENTRUST trials) recommend dual therapy only for selected patients with a (very) high risk of bleeding. It is, however, difficult to define patients with a high bleeding risk. Although there are several scores to assess bleeding risk (HAS-BLED for AF patients, CRUSADE for ACS patients, PRECISE-DAPT for PCI patients) [20–22], none is specifically suitable for AF patients undergoing PCI. On the other hand, there may be patients with a very high thrombotic risk for whom a longer period of triple therapy (>1 month) is advised by the current ESC guidelines. Similar to the bleeding risk, the thrombotic risk is difficult to define. It is also not clear how the currently available thromboembolic risk scores (CHA_2_DS_2_-VASc score and DAPT score) perform in these patients. Nevertheless, the data call for more patient-tailored antithrombotic management [23, 24].

For patients with chronic coronary syndrome and NSTEMI, roughly two-thirds of the interventional cardiologists would prescribe a full NOAC dose when the NOAC is combined with single or dual antiplatelet therapy. The practical guide on the use of NOACs suggests reducing the NOAC dose in case of a high bleeding risk in patients with concomitant antiplatelet therapy [25]. However, only dabigatran 110 mg twice daily has shown to be effective in stroke prevention; the reduced doses of other NOACs (rivaroxaban 15 mg once daily, apixaban 2.5 mg twice daily, edoxaban 30 mg once daily) have not. Hence, lowering the dose of NOACs might result in more thromboembolic events. In a recently published retrospective analysis, off-label use of the reduced dose of NOACs was associated with a 2.5 times increased risk of thromboembolism compared with warfarin [26].

A few studies have investigated the combination of a NOAC and antiplatelet therapy in patients with acute or chronic coronary syndrome without AF. In the APPRAISE‑2 trial, patients with recent ACS (median time 6 days) were randomly assigned to apixaban 5 mg twice daily on top of dual antiplatelet therapy or antiplatelet therapy alone. The addition of apixaban results in significantly more bleeding events, without less recurrent ischaemic events [27]. Although low-dose rivaroxaban (2.5 or 5 mg twice daily) on top of antiplatelet therapy leads to more bleeding in patients with ACS, this is counterbalanced by less cardiovascular events, including mortality [28]. In the large COMPASS trial studying patients with stable coronary artery disease, low-dose rivaroxaban (5 mg twice daily) plus acetylsalicylic acid resulted in an absolute risk reduction in cardiovascular death, stroke or nonfatal myocardial infarction, at the expense of more bleeding. The net clinical benefit was in favour of rivaroxaban on top of acetylsalicylic acid [29]. However, in the previously mentioned trials the studied NOAC doses were lower than those approved for stroke prevention in AF patients.

The most recent evidence comes from the AFIRE study. In this trial, patients with AF and chronic coronary syndrome who had undergone PCI or coronary artery bypass grafting at least 1 year before enrolment were randomised to rivaroxaban monotherapy (5 mg twice daily) or rivaroxaban (5 mg twice daily) plus a platelet inhibitor. Rivaroxaban monotherapy was noninferior to combination therapy for the composite primary endpoint of stroke, systemic embolism, myocardial infarction, unstable angina requiring revascularisation or death from any cause (hazard ratio 0.72, 95% confidence interval [CI] 0.55–0.95) and superior in terms of major bleeding according to the criteria of the International Society on Thrombosis and Haemostasis (hazard ratio 0.59, 95% CI 0.39–0.89) [30]. In our survey, the majority of the interventional cardiologists (>80%) would prescribe NOAC monotherapy 1 year after PCI.

### Study limitations

Our study has some limitations. Despite the high response rate (69.4%), nonresponders might reflect those with less interest in and knowledge of the topic (response bias). Furthermore, we did not invite all European interventional cardiologists, but only a selection. Therefore, this survey is representative of current clinical practice in the Netherlands, but not across Europe. Finally, as in most surveys, the study relied on self-report and we did not validate self-report against objective measures.

## Conclusion

In this online, international survey among interventional cardiologists, we observed heterogeneity in antithrombotic management strategies for AF patients treated with OAC undergoing PCI. This observed variety mirrors the heterogeneity of the many guidelines and consensus documents. Further research is needed to guide patient-tailored medicine for AF patients undergoing PCI.

## Caption Electronic Supplementary Material

Table 1 Overview of questions in the WOEST survey 2018
