# Supplementary material for: How cardiologists manage antithrombotic treatment of patients with atrial fibrillation undergoing percutaneous coronary stenting: the WOEST survey 2018
Source: Neth Heart J. 2020 Oct 14;29(3):135–41. doi: 10.1007/s12471-020-01500-3 (PMC7904986; doi:10.1007/s12471-020-01500-3)
Supplement: Supplementary file 1 — Table 1 Overview of questions in the WOEST survey 2018 [file 12471_2020_1500_MOESM1_ESM.docx]

**Tab. 1** Overview of questions in the WOEST survey 2018

| 1 | Is there a standardised protocol for the antithrombotic management of AF patients on VKA or NOAC who require PCI? | Yes, but only for patients on chronic VKA |
| --- | --- | --- |
|  |  | Yes, for patients on chronic VKA and NOAC |
|  |  | No, there is no protocol |
|  | **A male patient with AF using a standard dose NOAC admitted for NSTEMI requires PCI with stenting. The bleeding and thrombotic risks are intermediate. How would you treat this patient?** | |
| 2A | Would you initiate antiplatelet therapy immediately after admission? | Yes |
|  |  | No, I would start this during/after PCI |
| 2B | If yes, which antithrombotic regimen would you start? | ASA monotherapy (+ NOAC) |
|  |  | P2Y_12_ inhibitor monotherapy (+ NOAC) |
|  |  | Dual antiplatelet therapy (+ NOAC) |
| 3 | Would you discontinue the NOAC therapy before PCI? | Yes, without bridging |
|  |  | Yes, with bridging |
|  |  | No |
| 4 | Would you administer a bolus of heparin (UFH/LMWH) at the start of PCI when the patient is on NOAC? | Yes, the standard dose |
|  |  | Yes, the reduced dose |
|  |  | No |
| 5A | At discharge, what is your default antithrombotic strategy for a patient like this (intermediate bleeding risk and intermediate thrombotic risk)? | Triple therapy |
|  |  | Dual therapy |
| 5B | In case of a **high bleeding risk**, what is your default antithrombotic strategy at discharge? | Triple therapy |
|  |  | Dual therapy |
| 5C | In case of a **high bleeding risk *and* a high thrombotic risk**, what is your default antithrombotic strategy at discharge? | Triple therapy |
|  |  | Dual therapy |
| 6 | When you use triple therapy, for what period of time would you prescribe ASA? | 1 month |
|  |  | 3 months |
|  |  | 6 months |
|  |  | 12 months |
|  |  | >12 months |
| 7A | Which NOAC for stroke prevention would you prescribe at discharge? | I would continue the same NOAC as the patient was using before PCI |
|  |  | Dabigatran |
|  |  | Rivaroxaban |
|  |  | Apixaban |
|  |  | Edoxaban |
| 7B | Which dose would you prescribe at discharge? | The normal dose (NB For dabigatran both doses are tested for stroke prevention) |
|  |  | The reduced dose |
| 8 | If the patient was using a VKA instead of a NOAC, would you switch the patient to a NOAC after PCI? | Yes |
|  |  | No |
| 9 | Which antithrombotic treatment would you prescribe >1 year after PCI? | Monotherapy: NOAC |
|  |  | Dual therapy: NOAC and ASA or P2Y_12_ inhibitor |
|  | **The following questions are the same as the previous; however, they now concern a patient with AF using a standard NOAC dose undergoing elective PCI because of stable coronary artery disease. The bleeding and thrombotic risks are intermediate.** | |
| 10 | What antiplatelet therapy would you start before PCI? | ASA monotherapy (+ NOAC) |
|  |  | P2Y_12_ inhibitor monotherapy (+ NOAC) |
|  |  | Dual antiplatelet therapy (+ NOAC) |
| 11 | Would you discontinue the NOAC therapy before PCI? | Yes, without bridging |
|  |  | Yes, with bridging |
|  |  | No |
| 12 | Would you administer a bolus of heparin (UFH/LMWH) at the start of PCI when the patient is on NOAC? | Yes, the standard dose |
|  |  | Yes, the reduced dose |
|  |  | No |
| 13 | At discharge, what is your default antithrombotic strategy for a patient like this (intermediate bleeding risk and intermediate thrombotic risk)? | Triple therapy |
|  |  | Dual therapy |
| 14 | In case of a **high bleeding risk**, what is your default antithrombotic strategy at discharge? | Triple therapy |
|  |  | Dual therapy |
| 15 | In case of a **high bleeding risk *and* a high thrombotic risk**, what is your default antithrombotic strategy at discharge? | Triple therapy |
|  |  | Dual therapy |
| 16 | When you use triple therapy, for what period of time would you prescribe ASA? | 1 month |
|  |  | 3 months |
|  |  | 6 months |
|  |  | 12 months |
|  |  | >12 months |
| 17A | Which NOAC for stroke prevention would you prescribe at discharge? | I would continue the same NOAC as the patient was using before PCI |
|  |  | Dabigatran |
|  |  | Rivaroxaban |
|  |  | Apixaban |
|  |  | Edoxaban |
| 17B | Which dose would you prescribe at discharge? | The normal dose (NB For dabigatran both doses are tested for stroke prevention) |
|  |  | The reduced dose |
| 18 | If the patient was using a VKA instead of a NOAC, would you switch the patient to a NOAC after PCI? | Yes |
|  |  | No |
| 19 | Which antithrombotic treatment would you prescribe >1 year after PCI? | Monotherapy: NOAC |
|  |  | Dual therapy: NOAC and ASA or P2Y_12_ inhibitor |

*AF* atrial fibrillation, *VKA* vitamin K antagonist, *NOAC* non-vitamin K antagonist oral anticoagulant, *PCI* percutaneous coronary intervention, *NSTEMI* non-ST-elevation myocardial infarction, *ASA* acetylsalicylic acid, *UFH/LMWH* unfractionated heparin/low-molecular weight heparin
